# Adductor canal block versus periarticular infiltration for pain control following total knee arthroplasty: Study protocol for a randomized controlled trial

**DOI:** 10.1097/MD.0000000000019903

**Published:** 2020-04-24

**Authors:** Na Yuan, Jun Shi, Chunyan Lin, Jiang Li

**Affiliations:** Department of Anesthesiology, Ningbo No.6 Hospital, Zhejiang Province, China.

**Keywords:** adductor canal block, periarticular infiltration, randomized controlled trial, study protocol, total knee arthroplasty

## Abstract

**Background:**

Periarticular infiltration (PAI) and adductor canal block (ACB) have become popular modes of pain management after total knee arthroplasty. The purpose of our study is to evaluate the efficacy of ACB in comparison with PAI for pain control in patients undergoing primary total knee arthroplasty.

**Methods:**

This study is a prospective, 2-arm, parallel-group, open-label randomized controlled trial that is conducted at a single university hospital in China. A total of 120 patients who meet inclusion criteria are randomized in a ratio of 1:1 to either ACB or PAI group. The primary outcome is visual analog scale score at rest 24 hours after surgery, whereas the secondary outcomes include visual analog scale score at 48 hours after surgery, satisfaction, opioid consumption, and complications. All pain scores are assessed by an independent observer who is blinded to the allocation of groups.

**Results:**

This study has limited inclusion and exclusion criteria and a well-controlled intervention. This clinical trial is expected to provide evidence of better therapy for the pain management after total knee arthroplasty.

**Trial registration:**

This study protocol was registered in Research Registry (researchregistry5410).

## 1 Introduction

Over 600,000 total knee arthroplasties are performed each year in the United States.^[1]^ In the last decade, there has been a focus on multimodal postoperative pain management protocols, more rapid functional recovery, reduced length of hospital stay, and minimizing side effects of treatment while maintaining function.^[2]^ The widespread use of regional anesthesia in total knee arthroplasty has played a major positive role in these improvements.^[3]^ Femoral nerve blocks have been shown to reduce opioid consumption and decrease postoperative pain scores. In recent years, adductor canal block (ACB), at the midpart of the thigh, has gained favor over femoral nerve block, at the groin, with the benefit of maintaining a sensory block for pain control while minimizing motor blockade to the quadriceps/extensor mechanism.^[4]^ Greater motor block is typically seen with proximal femoral nerve blocks, which can hamper rehabilitation and increase the risk of falls.^[4]^ In addition to regional blocks, which are typically performed in the preoperative setting, some surgeons favor intraoperative periarticular infiltration (PAI), typically with bupivacaine, either in conjunction with an ACB or independently.^[5–9]^ In theory, PAI has the advantage of a sensory nerve block that is comparable with an ACB without the risks of quadriceps weakness, falls, and neurologic dysfunction.^[5–7,10]^

Utilization of these pain management tools in total knee arthroplasty is not consistent across the country. Surgeons who prefer PAI therapy over an ACB cite potential delays of surgery due to the administration of the ACB in the preoperative area, increased costs due to the ACB, and the small risks associated with a regional block. Alternatively, high-dose PAIs can convey risks of systemic and cardiovascular complications.^[11]^ In addition, advocates of regional blocks contend that ACBs have better consistency and predictability.

The purpose of this randomized controlled trial is to compare the efficacy of ACB and PAI for pain management in patients undergoing a total knee arthroplasty. We hypothesized that standard PAI would be as effective as ACB for postoperative pain management following total knee arthroplasty.

## 2 Material and method

### 2.1 Study design

**Figure 1:**
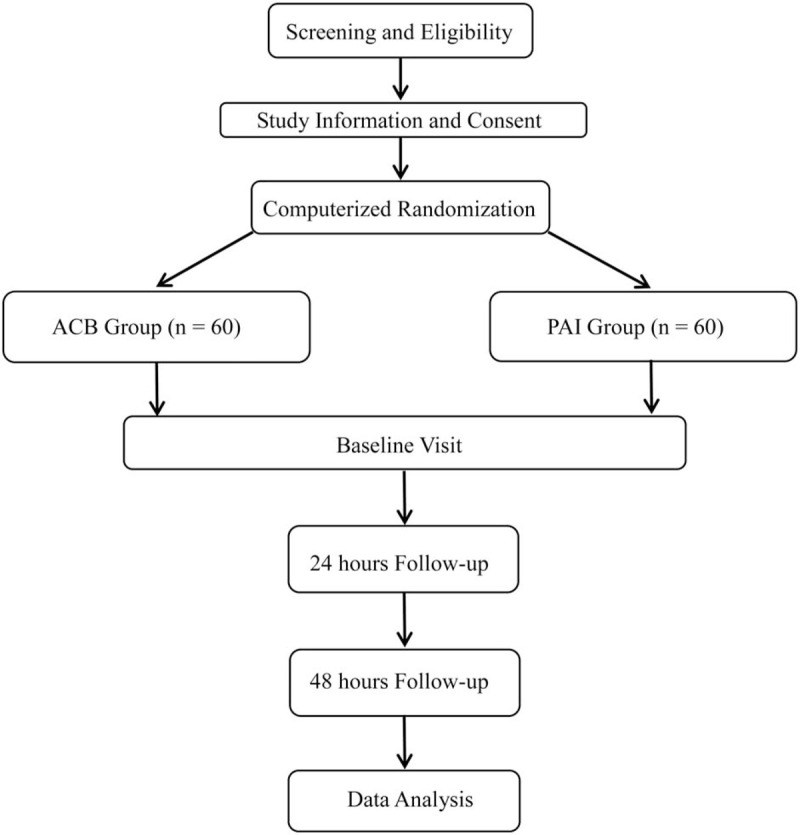
Flow diagram of the study.

This study is a prospective, 2-arm, parallel-group, open-label randomized controlled trial that is conducted at a single university hospital in China. The study is conducted at a single university hospital in China from March 1, 2020 till June 30, 2021. The study was approved by the institutional review board (CRD42019146454), and all patients provide written informed consent. Postoperative pain at rest after surgery is the focus of the study. This systematic review protocol has been subsequently registered in Research Registry (researchregistry5410). The flowchart of this trial is shown in Figure 1.

### 2.2 Participants

Eligible patients are scheduled for primary unilateral TKA, older than 18 years, and have the ability to cooperate with data acquisition. During the study period, all TKAs are performed as an in- hospital procedures. The exclusion criteria are the following: patients unwilling to participate, poorly controlled diabetes, history of inflammatory arthritis, nonambulatory/bed ridden patients, known allergy to the anesthetic drugs, history of bleeding disorder, history of arrhythmia or seizures, sepsis, and pre-existing lower extremity neurological abnormality. Participants are informed that the study is comparing the efficacy of ACB and PAI for pain control following primary unilateral TKA and that they are randomly assigned to either the ACB and PAI group.

### 2.3 Randomization

An independent operator not otherwise involved in the trial generate randomized numbers from 0 to 99 using computer software (Excel 2010; Microsoft, Redmond, WA). Each time a patient is included in the trial, the generated randomized number is assigned accordingly. The patients assigned an even number are allocated to the ACB group and those with an odd number are allocated to the PAI group.

### 2.4 Interventions

For group A (ACB), an ultrasound transducer is used to identify the adductor canal. The transducer locate the adductor canal at mid-thigh, halfway between the inguinal crease and patella. Superficial femoral artery, sartorius muscle, adductor longus muscle, and adductor magnus muscle are identified. The hyper echoic structure located anterolateral to the artery (saphenous nerve and nerve to vastus medialis) is identified as the target injection site. A 22-guage, 100-mm needle is introduced lateral to medial under ultrasound guidance using linear probe of a sonosite machine. Solution containing 30 mL of 0.5% ropivacaine and 100 mcg of clonidine (total volume = 30.7 mL) is injected after ensuring correct placement of the needle.

For group B (PAI), the solution contained local anesthetic agent (ropivacaine), NSAID (ketorolac), epinephrine (adrenaline), clonidine, and normal saline according to the weight of the patient and is injected using a 20-gauge spinal needle with 20 cc syringe. The PAI is given in 8 zones around the knee as shown in Table 1.

**Table 1:**
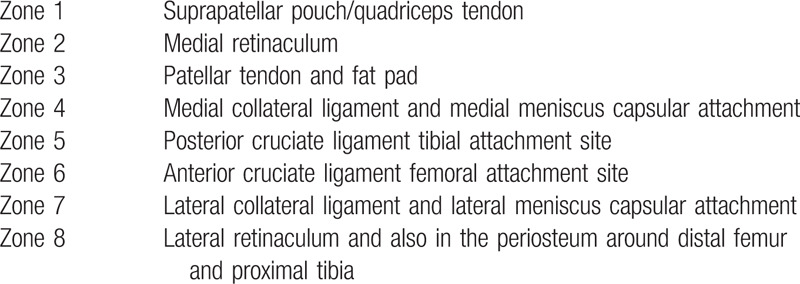
Zones for periarticular infiltration around knee.

### 2.5 Preoperative and postoperative medications

Intravenous patient-controlled analgesia (PCA) fentanyl citrate is used for patients complaining of intolerable postoperative pain. Intravenous PCA fentanyl is started when patients request to use it and continue at 20 m g/h. For additional rescue, intravenous PCA fentanyl is used (20 m g/dose; lockout time, 20 min). The PCA is discontinued 24 hours after surgery, whereas the PCA pump device record the total volume of fentanyl consumed. During the study period, intravenous PCA is routinely used as rescue analgesia for inpatient surgeries. From the day after surgery, an oral COX-2-selective nonsteroidal anti-inflammatory drug (200 mg of celecoxib) is administered 2 times a day until 14 days after surgery. No oral narcotic pain medications are used during the study period. Antibiotic prophylaxis with 1.5 g of sulbactam/ampicillin is intravenously administered 30 minutes before surgery and every 8 hours after surgery for 2 days.

### 2.6 Outcome measurements

The primary outcome is pain at rest 24 hours after surgery. Pain intensity is rated using a 100-mm horizontal VAS, for which 0 mm represent no pain and 100 mm represent extreme pain and compare between groups. The postoperative pain levels at rest other than at 24 hours after surgery are compared between groups. The postoperative VAS scores for patient satisfaction with pain management are compared between the groups until 3 days after surgery. Satisfaction levels are rated using a 100 mm horizontal VAS, for which 0 mm represent completely dissatisfied and 100 mm represent completely satisfied. The total amount of fentanyl consumption in postoperative intravenous PCA is evaluated by the PCA record. Any postoperative complications that occurred during the course of the trial are recorded. All pain scores are assessed by an independent observer who is blinded to the allocation of groups.

### 2.7 Sample size calculation

We estimate that with 50 participants in each group, the study will have more than 80% power to detect a clinically important difference between the groups in regard to the change in the pain score evaluated with the VAS. This is assuming a mean intergroup difference in score of 20 mm based on previous literature and a pooled standard deviation of 35 mm on the basis of preliminary data at an alpha level of 5%. Based on this estimation, a total of 120 patients are needed with an allowance for 10% drop-out.

### 2.8 Statistical analysis

The primary outcome of this study is compared between groups with a Student *t* test. For missing primary outcome data, VAS scores are replaced with the median scores for the other patients of the same treatment group at the same point in time. The comparisons between the study groups are performed with a chi-square test for categorical variables and a Student t test for continuous variables. All tests are 2-sided, and *P* < .05 is considered statistically significant.

## 3 Discussion

Patients undergoing total knee arthroplasty suffer from moderate to severe pain postoperatively. Although there have been advances in technologies and instrumentations in total knee arthroplasty, pain management after the operation is still evolving.^[12–14]^ Various methods of pain control used in the previous years include epidural analgesia, femoral nerve block, PAI, and systemic analgesia. Perioperative pain management with PAI is a safe and effective method of controlling pain after total knee arthroplasty and it also eliminates the risk associated with FNB of quadriceps weakness. Effective use of PAI requires specific knowledge of the relevant neuroanatomy of the knee. PAI contains cocktail of local anesthetics, non steroidal anti inflammatory drugs, epinephrine (adrenaline), and normal salinewhich isinjected intothe periarticular tissues around the knee joint during the operation. It has gained popularity for its simplicity, safety, and selective sensory blockade unlike the motor blockade associated with femoral nerve block and epidural analgesia.^[9,15]^

In the recent years, ultrasound-guided ACB has gained popularity over femoral nerve block for management of pain in total knee arthroplasty patients. The adductor canal (also known as the sub-sartorial or the Hunter's canal) is located within the middle third of the anterior-medial thigh and extends from the apex of the femoral triangle to the adductor hiatus. The contents of the adductor canal have traditionally been described as the femoral artery and vein, 2 fascicular branches of the femoral nerve, the saphenous nerve and the nerve to the vastus medialis, and the articular contribution of the obturator nerve, which enters the distal adductor canal just proximal to the adductor hiatus.^[11,16]^ The ACB is a sensory nerve block with some effect on the motor function of vastus medialis as the motor branch passes through the adductor canal. Isolated and partial effect on motor weakness of vastus medialis decreases the tendency of fall while walking. Use of ACB needs ultrasound and does not provide pain relief at the posterior aspect of the knee.^[13,17]^ Whether PAI offers better pain control than ACB after total knee arthroplasty remains controversial. Therefore, this study is conducted to resolve this issue.

This trial has some limitations. First, the subjects may be exclusively Chinese. Therefore, the data from this clinical trial cannot be applied to other ethnic groups. Second, owing to the small sample size, the results of this study cannot be generalized. Despite these limitations, this trial is expected to provide evidence of better therapy for the pain management after total knee arthroplasty.

## Author contributions

Jun Shi and Na Yuan planned the study design and wrote the study protocol. Chunyan Lin and Jiang Li reviewed the study protocol. Na Yuan, Jun Shi, and Chunyan Lin will recruit participants and collect data. Jun Shi and Na Yuan wrote the manuscript. All of the authors have read, commented on, and contributed to the submitted manuscript.

Jun Shi orcid: 0000-0002-5768-5560.
